# Relationship between bone resorption and sclerostin regulation in apical periodontitis lesions

**DOI:** 10.1007/s10266-025-01118-0

**Published:** 2025-05-05

**Authors:** Ebru Uysal, Seyda Ersahan, Fatih Ozcelik, Yelda Erdem Hepsenoglu

**Affiliations:** 1 Department of Endodontics, Faculty of Dentistry, Istanbul Medipol University, Istanbul, Turkey; 2 Department of Medical Biochemistry, Sisli Hamidiye Etfal Training and Research Hospital, University of Health Sciences Turkiye, Istanbul, Turkey

**Keywords:** Apical periodontitis, Bone resorption, Gingival crevicular fluid, Sclerostin, PGE2

## Abstract

This study examined the relationship between gingival crevicular fluid (GCF) and serum sclerostin and PGE2 levels and the inflammatory bone resorption associated with chronic apical periodontitis (AP) as well as the correlation between sclerostin regulation and RANKL and MMP-9 levels. Ninety participants were divided into three groups based on PAI scores, as follows: Group 1 (control group, PAI 1–2, *n*:35); Group 2 (PAI 3–4, *n*:35); Group 3 (PAI 5 in at least 1 tooth, *n*:55). Sclerostin, PGE2, RANKL, and MMP-9 levels were measured in the serum and GCF of all participants. GCF sclerostin, RANKL, and PGE2 levels of Group 3 were significantly higher than those of Groups 1 and 2 (75.8 ± 43.3, 37.0 ± 6.4 and 42.7 ± 8.2 ng/mL, *p* < 0.0001; 319 ± 167, 244 ± 41 and 248 ± 49 ng/L, *p* = 0.0029; 193 ± 87, 141 ± 90 and 137 ± 79 ng/L, *p* = 0.0028, respectively for Groups 3, 2, and 1). GCF MMP-9 levels of Group 3 were significantly higher than those of Group 1 (465 ± 162 and 384 ± 44 ng/mL, *p* = 0.0340). Group 3 also had elevated serum sclerostin and PGE-2 levels, but the differences between groups were less pronounced in serum than in GCF (*p* < 0.05). In the ROC analysis performed for the diagnostic performance of abscess formation in AP, the sensitivity of the GCF sclerostin and GCF PGE2 tests was determined as 65.5% and 72.7%, specificity as 98.6% and 68.6%, and AUC as 0.768 and 0.712, respectively (*p* < 0.0001). Both GCF sclerostin and PGE-2 independently showed close relationships with PAI-abscess scores used to determine AP severity and they can be used in combination for diagnosing and monitoring AP-related bone resorption.

## Introduction

Chronic apical periodontitis (AP) is defined as the persistent infection of periapical tissue occurring when dental pulp is invaded by pathogenic microorganisms as a result of trauma or iatrogenesis. Progression of this inflammatory disease is characterized by loss of periodontal tissue and alveolar bone. Because AP usually progresses asymptomatically, it is often detected only during routine radiographic examination, the findings of which are insufficient for accurately determining AP severity. Up until now, the use of inflammatory markers in routine clinical evaluations has been quite limited. Considering that the main mechanism behind AP pathogenesis is the inflammatory process triggered by microbial factors, developing a new periapical scoring system that includes inflammatory markers could help improve the accuracy of AP diagnosis.

Studies have demonstrated that biologically active factors including bacterial products, host cells, and locally produced cytokines play important roles in AP pathogenesis [1]. Recent research has aimed to better understand the pathogenesis of periodontitis by focusing on individual mediators and cytokines specifically involved in bone destruction [2]. Sclerostin is a glycoprotein with a molecular weight of 40 kDa composed of 213 amino acids that is generally synthesized by osteocytes and to a lesser extent by chondrocytes and osteoclasts. Sclerostin synthesis has been observed in alveolar bone osteocytes, dental pulp odontoblasts, stem cells, and cementum cementocytes as well as in periodontal tissue and gingival crevicular fluid. Sclerostin synthesis is affected by oxygen, hormones, transcription factors, orthodontic force, and mechanical stress [3, 4]. Through its role in the proliferation, differentiation, and apoptosis of preosteoblastic cells and osteoblasts, sclerostin regulates bone remodeling processes and reduces bone mineralization in all bones [3, 5]. Sclerostin has been shown to act as a potent inhibitor of osteogenesis. Studies have suggested that sclerostin is responsible for blocking the Wnt/β-catenin signaling pathway that is crucial for skeletal development and down-regulating the bone morphogenetic proteins (BMPs) responsible for bone formation, thus giving rise to the suggestion that sclerostin should be considered a BMP antagonist [6, 7]. Sclerostin has also been shown to play a critical role in bone resorption by up-regulating expression of the receptor activator nuclear factor-κB ligand (RANKL), which is involved in osteoclast differentiation and activation, and down-regulating expression of osteoprotegerin (OPG), a soluble RANKL decoy receptor [8, 9] that inhibits bone destruction by osteoclasts [10, 11]. By regulating the RANKL/OPG axis, sclerostin increases RANKL expression and down-regulates OPG expression. Thus, it supports osteoclast formation and osteocyte activity [8, 9]. Although all this information shows that the role of sclerostin in bone metabolism is well explained, its specific relationship with apical periodontitis (AP) has not yet been fully emphasized. In this context, a study on this subject has the potential to fill an important gap.

Wnt/β-catenin signaling pathway is a pathway that has a critical role in embryonic development and tissue homeostasis. It has been reported that disruption of the Wnt/β-catenin pathway may be involved in the pathogenesis of many serious diseases, including cancer. For this reason, compounds targeting the Wnt signaling pathway have been considered for use in treatment [12]. Sclerostin interferes with the Wnt/β-catenin signaling pathway by competitively binding to the lipoprotein-like receptor 5/6, thereby preventing the signal from proceeding to the β-catenin pathway and thus impairing the osteogenic function of the osteoblasts [12]. It has also been suggested that while sclerostin increases the production of RANKL, it also reduces the production of OPG by osteoblasts and pre-osteoblasts, which inhibits bone destruction by osteoclasts [10, 11]. The understanding that sclerostin blocks the Wnt/β-catenin signaling pathway via BMPs [6, 7], its association with oral diseases such as periodontitis and pulpitis, and its selection as a therapeutic target in studies aimed at establishing effective treatment methods [10] has made it the subject of current research for more detailed evidence. Nowadays, research on mediators and cytokines involved in bone destruction continues rapidly in order to understand the pathogenesis of periodontal disease [2]. In this context, we also focused the contribution of sclerostin, which suppresses bone metabolism via the Wnt/β-catenin pathway in the pathogenesis of AP, which is characterized by inflammatory process and bone destruction, to the bone resorption process in AP.

Most recently, studies have shown that bone formation and destruction are governed by a series of cytokines related to the TNF family [13–15]. These molecules appear to be the last-step effectors of osteotropic factors such as RANK–RANKL interaction, which is a stimulator of bone resorption, and OPG, which acts as a protector [14]. High levels of interleukin-1 (IL-1), interleukin-6 (IL-6), prostaglandin E2 (PGE2), and TNF-α have been detected in the gingiva and gingival crevicular fluid (GCF) of patients with periodontal disease [16, 17]. Inhibition of COX enzymes and antagonism of PGE2 are of great therapeutic interest, as PGE2 is involved in many biological functions, such as the regulation of immune responses and inflammation in physiological and pathological conditions [18]. Therefore, PGE2 was preferred over other markers in this study. Furthermore, this apparent indication that locally active inflammatory cytokines play an active role in the formation of periodontitis-related pathologies suggests that in addition to systemic blood cytokine levels, locally produced cytokine levels may be useful in the diagnosis and follow-up of AP and other periodontal diseases.

Matrix metalloproteinase-9 (MMP-9), which can be expressed by neutrophils, macrophages, T cells, mast cells, and other odontoblasts [19], can degrade various types of collagens, including several forms of elastin. MMP-9 has also been shown to be involved in the pathogenesis of pulp and periapical inflammation and has been detected in intracanal samples in teeth with apical abscesses [20] and in high amounts in the GCF of teeth with periapical lesions [21]. Considering that high levels of MMP-9 may be related to IL-1α, TNF-α, and bacterial LPS stimulation, the measurement of GCF MMP-9 levels has been suggested as a means of monitoring chronic apical periodontitis [22]. Since MMP-9 contributes to the progression of inflammation in periodontal and apical tissues by degrading collagen and other extracellular matrix components, it was thought that it could be a suitable marker for understanding the pathological processes of AP, which progresses with bone resorption in advanced stages.

Radiological imaging is currently an important tool for the diagnosis of apical periodontitis. The current approaches place the use of inflammatory markers at the forefront in determining the severity of tissue damage and disease. This study investigated the relationship between local and systemic sclerostin levels and the severity of AP-related inflammatory bone resorption as well as the findings of radiographic imaging. It also examined the metabolic relationship between sclerostin regulation and RANKL, MMP-9, and PGE-2.

## Materials and methods

### Trial design and ethical approval

This single-centered, cross-sectional analytical study was conducted at the Endodontics Department of Istanbul Medipol University and included assessments of patients with AP conducted between August 2023 and January 2024. Ethical approval was obtained from the Institutional Ethical Committee (Istanbul Medipol University, Clinical Research Ethics Committee, Decision Number: E-10840098-202.3.02-118/623, 27/07/2023), and written consent was obtained from all participants. All procedures complied with the 1964 Helsinki declaration.

### Inclusion and exclusion criteria

A total of 90 patients diagnosed with chronic AP [(Group 2 (PAI 3–4, n:35) and Group 3 (PAI 5 in at least 1 tooth, n:55)] were selected from among approximately 800 patients applying to the Endodontics Department for routine control examinations. A gender-, age- and weight-matched control group (Group 1: PAI 1–2) comprised 35 oral and systemically healthy individuals applying to the clinic for routine check-ups that included routine examinations. The total number of participants included in this study was 125 people. Exclusion criteria included the following: acute or chronic inflammatory/rheumatoid, cardiovascular, muscle-joint-bone, or connective tissue diseases or diabetes mellitus; local or generalized infections; a history of smoking, antibiotic or anti-inflammatory drug use within the past 6 months; high doses of biotin within the past 48 h; and pregnancy/lactation. Patients undergoing dental treatment and those with another periodontal disease in addition to AP were also excluded from this study.

### Demographic and clinical features

Demographic characteristics of the participants [age, body mass index (BMI), the Simplified Oral Hygiene Index (OHI-S)], findings of intraoral examination [number of teeth with root canal treatment (RCT), dental crown, composite/amalgam fillings (CF), and number of missing teeth (NMT), etc.] and radiological findings [periapical index (PAI) score] were recorded. The oral hygiene status of participants was evaluated by the Simplified Oral Hygiene Index (OHI-S) [23].

### Apical periodontitis classification and abscess scoring based on the PAI system

Radiographs were taken of all teeth present in the oral cavity and assessed for the presence of radiolucent images associated with the periapical region and radiographic bone loss. Panoramic radiographs were obtained using a digital panoramic unit (VistaPano S, Durr Dental AG, Germany) operated at 73kVp and 10 mA in standard mode and an exposure time of 13,500 ms. Periapical radiographs were obtained from a Carestream RVG 5200 (RVG; Carestream Health Inc, Atlanta, CA, USA) system with an X-ray unit operated at 70 kV, 8 mA, and using the bisecting angle technique. Radiographs were analyzed with Kodak Dental Imaging Software. The presence of periapical radiolucency without periodontal disease (probing depth of < 3 mm and no radiographic bone loss) was considered sufficient for a diagnosis of AP. Participants were divided into three groups based on periapical index (PAI) scores suggested by Ørstavik et al. [24], as follows: Group 1 (control group, health patients without AP): 35 healthy individuals with PAI score 1–2; Group 2 (mild-moderate AP): 35 patients with AP and PAI score 3–4; Group 3 (severe AP): 55 patients with AP and PAI score 5 (characterized by bone resorption). A further classification named abscess scoring based on the periapical index (AS-PAI) was performed as an indicator of disease progression using the PAI system. Participants were divided into three subgroups according to the highest AS-PAI score of any tooth, as follows: AS-PAI 0: those having PAI score below 5 for all examined teeth, includes the cases in both Group 1 and Group2, n:70; AS-PAI 1: those with PAI score of 5 in only one tooth, n:31; and AS-PAI 2: those with a PAI score of 5 in two or more teeth, n:24. While the patients in Group 1 and Group 2 were classified as AS-PAI 0 (PAI < 5), the patients in Group 3 were divided into either AS-PAI 1 or AS-PAI 2 depending on the number of teeth having a PAI 5 score. Serum and GCF of all participants were collected and analyzed to determine sclerostin, RANKL, MMP-9, and PGE2 levels.

### Venous blood collection

Fasting (8–10 h) venous blood was obtained from forearm antecubital/basic veins of all participants and kept at room temperature for 30 min. The samples were centrifuged at 2500 xg for 10 min, and the upper layer of sera was separated. In order to prevent optical interference in the biochemistry autoanalyzer device (Cobas 8000 Chemistry Analyzer, USA), Hemolysis Indices (HIs) of sera were obtained, and samples with an HI of ˃50 mg/dl Hb were excluded from this study. The remaining sera were aliquoted into 0.5 mL tubes (Eppendorf, Hamburg, Germany) and stored at -80 °C until analysis.

### GCF collection

GCF was collected from the area adjacent to the tooth with the highest AS-PAI score in each patient or the corresponding area in controls. Samples were taken prior to periodontal probing in order to avoid blood contamination, and patients were also asked not to eat or drink anything for at least 30 min before the procedure. Samples were taken from the mesial, distal, and buccal surfaces of the selected tooth using PerioPaper strips (OraFlow Inc., NY, USA). After isolating the sampling area with cotton rolls, plaque was removed, the area was gently air-dried, and a strip was inserted until a slight resistance was felt and kept in place for 30 s [24]. Sampling strips were then placed in Eppendorf tubes containing 100 µl of PBS and mixed for 15–20 s using a vortex device (Heidolph Reax Top Vortex, Schwabach, Germany) to allow the GCF to pass into PBS. Any strips contaminated with blood were excluded. GCF sample weights were measured using a precision balance (Shimadzu Libror, Model AEG-220, Germany) and recorded. Eppendorf tubes containing 100 µl PBS and empty PerioPaper strips were weighed on a precision balance and tared by removing them from the PerioPaper strips soaked in GCF. Tubes were stored at −80 °C until analysis. Prior to analysis, both GCF and serum samples were allowed to thaw slowly at + 4 °C and then brought to room temperature before measurement.

### ELISA analysis of biochemical parameters

Enzyme-linked immunosorbent assays (ELISA) were performed to measure serum sclerostin, RANKL, MMP-9, and PGE2 levels using a Microplate ELISA Reader (BioTek Epoch 2 Microplate ELISA Reader, USA). Sclerostin, RANKL, MMP-9 (Sunred Biological Technology Co., Ltd, Shanghai, China), and PGE2 test kits (Bioassay Technology Laboratory, Zhejiang, China) had sensitivity values of 0.175 ng/mL, 0.945 ng/L, 1.852 ng/ml, and 1.28 ng/L, respectively, and measuring ranges of 0.2–60 ng/mL, 1–300 ng/L, 2–600 ng/ml, and 2–600 ng/L, respectively. Intra- and inter-assay CVs of all tests were < 10%.

Biotinylated human sclerostin, RANKL, MMP-9, and PGE2 antibodies were added to pre-coated plate wells to bind with the sclerostin, RANKL, MMP-9, and PGE2 present in the samples. Streptavidin-HRP (Horseradish Peroxidase), which binds to biotinylated antibodies, was added to the wells, and the plates were incubated according to the manufacturers’ instructions. Wells were washed to remove unbound Streptavidin-HRPs. A peroxidase substrate was then added to the wells, producing a reaction between the substrate and the peroxidase enzyme that was terminated by the addition of an acidic stop solution. The intensity of the resulting color change is proportional to the levels of sclerostin, RANKL, MMP-9, and PGE2. Absorbance was measured at 450 nm.

### Statistical analysis

Statistical analysis of the study data was performed using the statistical software program SPSS (IBM Corp, 25 Version, Chicago, USA). Chi-square tests were used to evaluate categorical data (e.g., age, gender, number of teeth), and Kolmogorov–Smirnov tests were used to determine normality of distribution. Multi-group comparisons of nonparametric and parametric data were made using Kruskal–Wallis and one-way ANOVA tests, respectively. Relationships between independent variables were examined by Spearman (nonparametric data) and Pearson (parametric data) correlation analyses. Receiver operating characteristic (ROC) analysis was performed to determine the diagnostic performance of GCF sclerostin, GCF RANKL, GCF MMP-9, and GCF PGE2 tests in determining the severity of AP disease. The Area Under the Curve (AUC) was considered a practical measure of diagnostic adequacy, with an AUC value > 0.70 accepted as the minimum discriminatory power cutoff. AUC values of 0.8–0.9 and > 0.9 were considered to represent, respectively, “high” and “very high” discriminatory ability [26–28]. Fisher's Exact Test was used to determine the association between sclerostin, MMP-9, and PGE2 and severe abscess formation. Binary regression analysis was used to determine the effects of the independent variables sclerostin, RANKL, MMP-9, and PGE2 on the dependent variable severe AP and to predict AP severity. Bar plots provide graphic displays of nonparametric data. 

### Power analysis

According to a priori power analysis conducted by Kumar et al. [29] for their study of CGF PGE2 levels in patients with periodontal disease, at least 3 experimental subjects and 3 independent controls are required to assess GCF PGE2 levels (G*Power Version 3.0.10, effect size 3.42, α = 0.05, power = 0.90). However, Maïmoun et al. [30] stated that analysis of sclerostin levels in acute spinal cord injuries (G Power 3.1.9.4, effect size d: 0.6342, α err: 0.05, power: 0.80) requires at least 32 patients and 32 controls. Given that parametric tests conducted with a sample size of at least 30 offer stronger predictive power, this study was conducted with 35 subjects in both Group 1 (control group) and Group 2 (moderate AP) and 55 subjects in Group 3 (severe AP). Since Group 3 (PAI 5, *n* = 55), which represents severe cases in our study, includes more participants compared to other groups, this may increase the statistical power of the study results and enable more generalizable judgments to be reached.

## Results

### Comparison of demographic and clinical features

Demographic characteristics of the study groups are given in Table 1. Gender ratios, mean patient age, and age range were as follows: Group 1 (controls): M/F: 18/17, 32 ± 11 (23–61) years; Group 2: M/F: 20/15, 36 ± 11 (17–60) years; and Group 3: M/F: 26/29, 32 ± 12 (18–62) years. Mean BMI values for Groups 1, 2, and 3 were 26.1 ± 3.4 (21–35), 27.0 ± 4.7 (18–37), and 27.0 ± 4.2 (19–37) kg/m^2^, respectively. No differences in gender, age, or BMI were observed among the groups (*p* > 0.05); therefore, the differences observed among groups in other parameters were considered to be independent of these three demographic characteristics.

**Table 1:** Comparison of demographic characteristics and clinical findings of the study groups

	Group 1 (PAI 1–2)	Group 2 (PAI 3–4)	Group 3 (PAI 5)	*p* value
*N*	35	35	55	–
Gender. M (%)	18(%51)	20(%57)	26(%47)	^c^0.3800
Age. years	32.4 ± 10.9	35.6 ± 10.8	31.9 ± 12.0	^b^0.2761
BMI. kg/m^2^	26.1 ± 3.4	27.0 ± 4.7	27.0 ± 4.2	^b^0.5329
OHI score	1.2 ± 0.4 1.1(0.6–2.0)	4.7 ± 1.1 4.8(2.3–6.7)	4.7 ± 1.0 4.8(1.9–6.3)	^b^< 0.0001
Intergroup *p*	< 0.001	< 0.001	> 0.05	
AS-PAI score	0	0	1.4 ± 0.5 1(1–2)	^a^< 0.0001
Intergroup *p*	> 0.05	< 0.001	< 0.001	
Number of RCT. n	0.9 ± 1.2 0.0(0.0–5.0)	1.8 ± 2.1 1.0(0.0–9.0)	1.9 ± 2.1 1.0(0.0–10.0)	^a^0.0123
Intergroup p	> 0.05	< 0.05	> 0.05	
Number of Crown. n	0.9 ± 3.1 0.0(0.0–15.0)	2.3 ± 3.8 0.0(0.0–12.0)	2.2 ± 4.2 0.0(0.0–18.0)	^a^0.0194
Intergroup *p*	< 0.05	< 0.05	> 0.05	
Number of fillings. *n*	1.8 ± 2.0 1.0(0.0–9.0)	4.1 ± 3.1 4.0(0.0–11.0)	4.2 ± 3.7 3.0(0.0–17.0)	^b^0.0008
Intergroup *p*	< 0.01	< 0.01	> 0.05	
Number of missing teeth. *n*	0.8 ± 2.2 0.0(0.0–12.0)	3.3 ± 3.7 2.0(0.0–16.0)	3.0 ± 4.1 2.0(0.0–20.0)	^a^< 0.0001
	< 0.001	< 0.001	> 0.05	

^a^Kruskal–Wallis Test (Nonparametric ANOVA)

^b^One-way ANOVA test

^c^Chi-Square test

Statistical significance = *p* < 0.05

Parametric data are shown as mean ± standard deviation

PAI 1–2: Control group. M: Male. BMI: Body mass index, AS-PAI: Abscess scoring based on the periapical index, AS-PAI 0: PAI < 5, AS-PAI 1: only 1 tooth with PAI 5, AS-PAI 2: > 2 tooth with PAI 5, RCT: Root canal treatment, OHI: Oral hygiene index

Results of one-way ANOVA and Kruskal–Wallis analysis of clinical and radiographic findings are given in Table 1. No significant differences were observed in the OHI scores of Groups 2 and 3; however, OHI scores of both Groups 2 and 3 were significantly higher than those of Group 1 (controls) (*p* < 0.0001). Group 3 also had an AS-PAI score that was significantly higher than those of both Groups 1 and 2 (*p* < 0.0001) and a significantly higher number of teeth with root canal treatment than Group 1 (*p* = 0.0123). While there were no statistical differences in the numbers of crowns, dental fillings, and missing teeth in Groups 2 and 3 (*p* > 0.05), both groups had significantly higher numbers of crowns, dental fillings, and missing teeth than Group 1 (*p* < 0.05).

### Comparison of biochemical parameters

**Fig. 1:**
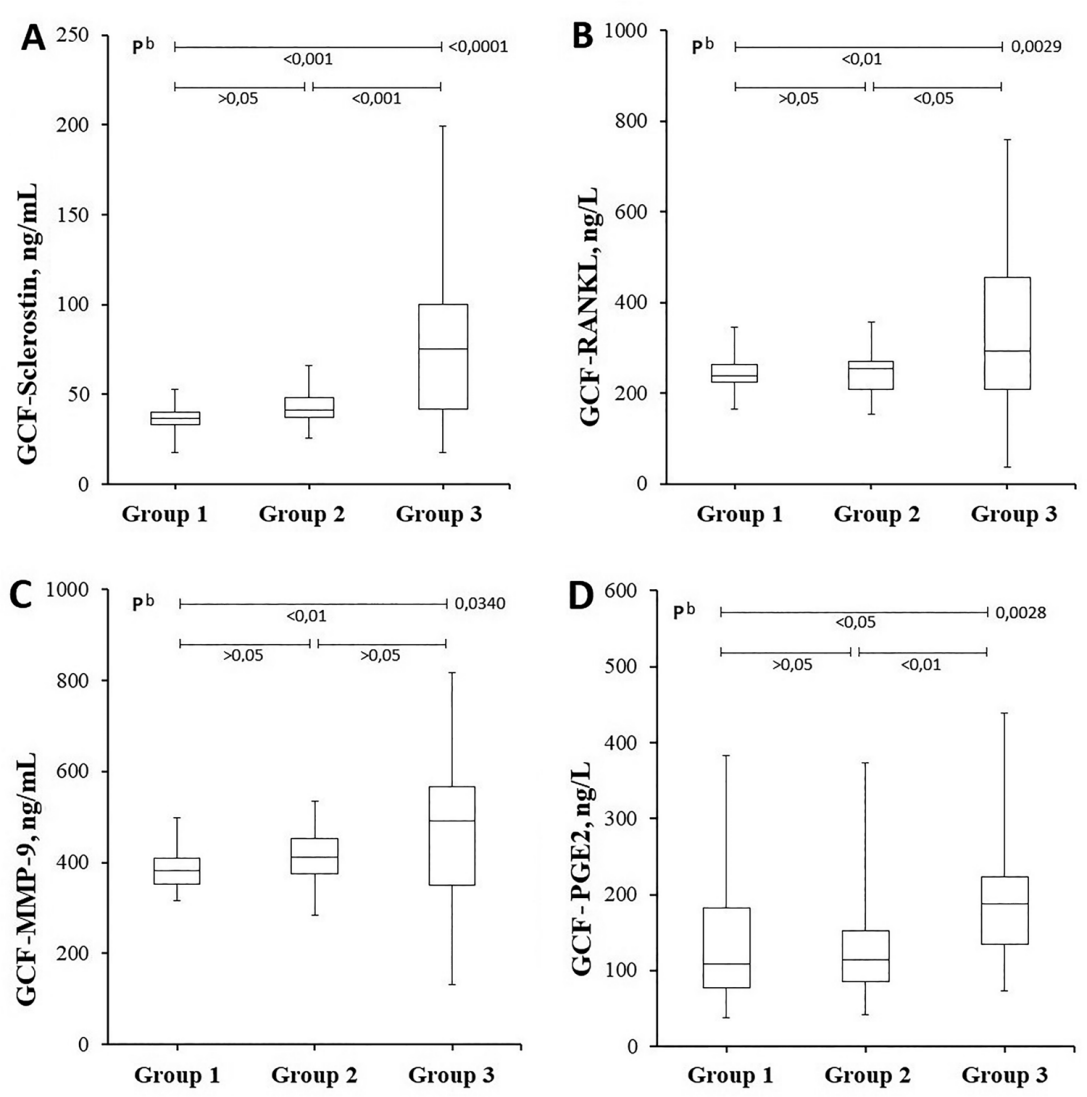
Gingival crevicular fluid levels of sclerostin, RANKL, MMP-9, and PGE2

Results of GCF biochemical analysis are shown in Table 2 and Fig. 1. GCF sclerostin levels were significantly higher in Group 3 (patients with severe AP and higher AS-PAI scores) as compared to Groups 1 and 2 (75.8 ± 43.3 vs. 37.0 ± 6.4 and 42.7 ± 8.2 ng/mL; *p* < 0.001, respectively, for Groups 3, 2, and 1), but did not vary significantly between Groups 1 and 2 (*p* > 0.05). Similarly, Group 3 had significantly higher GCF RANKL levels (319 ± 167 vs. 244 ± 41 and 248 ± 49 ng/L; p < 0.05, respectively, for Groups 3, 2, and 1) and GCF PGE2 levels (193 ± 87 vs. 141 ± 90 and 137 ± 79 ng/L; *p* < 0.05, respectively, for Groups 3, 2, and 1) as compared to both Groups 1 and 2, whereas these levels did not vary significantly between Groups 1 and 2 (*p* > 0.05). GCF MMP-9 levels were also significantly higher (465 ± 162 vs. 384 ± 44 ng/mL, *p* < 0.01) in Group 3 as compared to Group 1 (controls).

**Table 2:** Comparison of gingival crevicular fluid biochemical parameters of the study groups

	Group 1 (PAI 1–2)	Group 2 (PAI 3–4)	Group 3 (PAI 5)	*p* value
n	35	35	55	–
GCF sclerostin, ng/mL	37.0 ± 6.4	42.7 ± 8.2	75.8 ± 43.3	^b^< 0.0001
Intergroup *p*	> 0.05	< 0.001	< 0.001	
GCF RANKL, ng/L	244 ± 41	248 ± 49	319 ± 167	^b^0.0029
Intergroup *p*	> 0.05	< 0.01	< 0.05	
GCF MMP-9, ng/mL	384 ± 44	409 ± 55	465 ± 162	^b^0.0340
Intergroup *p*	> 0.05	< 0.01	> 0.05	
GCF PGE2, ng/L	141 ± 90	137 ± 79	193 ± 87	^b^0.0028
Intergroup *p*	> 0.05	< 0.05	< 0.01	

^a^Kruskal–Wallis Test (Nonparametric ANOVA)

^b^One-way Analysis of Variance (ANOVA)

Statistical significance = *p* < 0.05. When the p values obtained with ANOVA tests are found to be < 0.05, *p* values between the groups (comparison *p*) are determined (Group 1–2, Group 1–3, and Group 2–3, respectively)

Parametric data are shown as mean ± standard deviation

GCF: gingival crevicular fluid, RANKL: Receptor activator of NF-κB ligand, MMP-9: Matrix metalloproteinase-9, PGE2: Prostaglandin E2

**Fig. 2:**
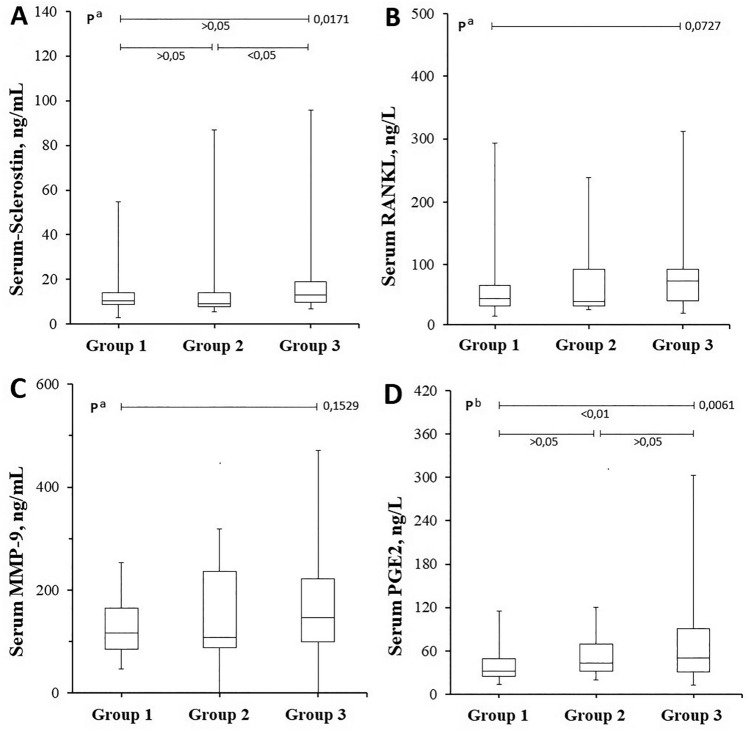
Serum levels of sclerostin, RANKL, MMP-9, and PGE2

Results of serum biochemical analysis are shown in Table 3 and Fig. 2. Serum sclerostin levels of Group 3 were significantly higher (*p* < 0.05) than those of both Group 1 and Group 2, whereas the difference in the serum sclerostin levels of Groups 1 and 2 was not statistically significant (*p* > 0.05). Serum PGE2 levels were significantly higher in Group 3 as compared to Group 1 (*p* < 0.05) (Fig. 2). Serum RANKL and MMP-9 levels did not vary significantly among groups (*p* > 0.05).

**Table 3:** Comparison of serum biochemical parameters of the study groups

	Group 1 (PAI 1–2)	Group 2 (PAI 3–4)	Group 3 (PAI 5)	*p* value
n	35	35	55	–
s-Sclerostin. ng/mL	15.1 ± 11.8 10.3(3.0–54.8)	15.8 ± 16.0 9.2(5.6–86.8)	18.6 ± 15.9 12.9(6.8–96.0)	^a^0.0171
Intergroup *p*	> 0.05	> 0.05	< 0.05	
s-RANKL. ng/L	66.4 ± 57.9 46.4(14.0–293.4)	75.8 ± 64.1 42.6(29.8–238)	79.8 ± 54.9 73.9(17.9–312)	^a^0.0727
s-MMP-9. ng/mL	152 ± 102 116(51–510)	178 ± 138 108(68–518)	184 ± 110 146(70–545)	^a^0.1529
s-PGE2. ng/L	39.9 ± 25.4 31.5(13.5–114.8)	51.5 ± 27.7 42.9(20.2–120)	69.2 ± 56.0 50.2(13.1–303)	^b^0.0061
Intergroup *p*	> 0.05	< 0.01	> 0.05	

^a^Kruskal–Wallis Test (Nonparametric ANOVA)

^b^One-way Analysis of Variance (ANOVA)

^c^Chi-Square test

Statistical significance = *p* < 0.05. When the *p* values obtained with ANOVA tests are found to be < 0.05, *p* values between the groups (comparison *p*) are determined (Group 1–2, Group 1–3, and Group 2–3, respectively)

Parametric data are shown as mean ± standard deviation. Nonparametric data are shown as mean ± standard deviation and median (min–max)

RANKL: Receptor activator of NF-κB ligand, MMP-9: Matrix metalloproteinase-9, PGE2: Prostaglandin E2, s: Serum

Correlation matrix analysis showed good to moderate correlation between PAI-AP severity and PAI-abscess score (*r* = 0.811), OHI score (*r* = 0.762), and GCF sclerostin (*r* = 0.496); moderate to low correlation between PAI-abscess score and OHI-S (*r* = 0.495), GCF sclerostin (*r* = 0.476), and GCF RANKL (*r* = 0.300); and low, but statistically significant, correlation between GCF sclerostin and GCF RANKL levels (*r* = 0.476). When the correlations detected in the correlation matrix analysis were re-analyzed with appropriate statistical methods depending on whether the data were parametric or nonparametric, it was determined that the correlation between PAI-AP severity and AS-PAI score and between PAI-AP severity and OHI score was a good positive correlation (Spearman r (rs) = 0.884, 95% CI: 0.8376 to 0.9184; *p* < 0.0001 and rs = 0.429, 95% CI: 0.269 to 0.566; *p* < 0.0001).

**Fig. 3:**
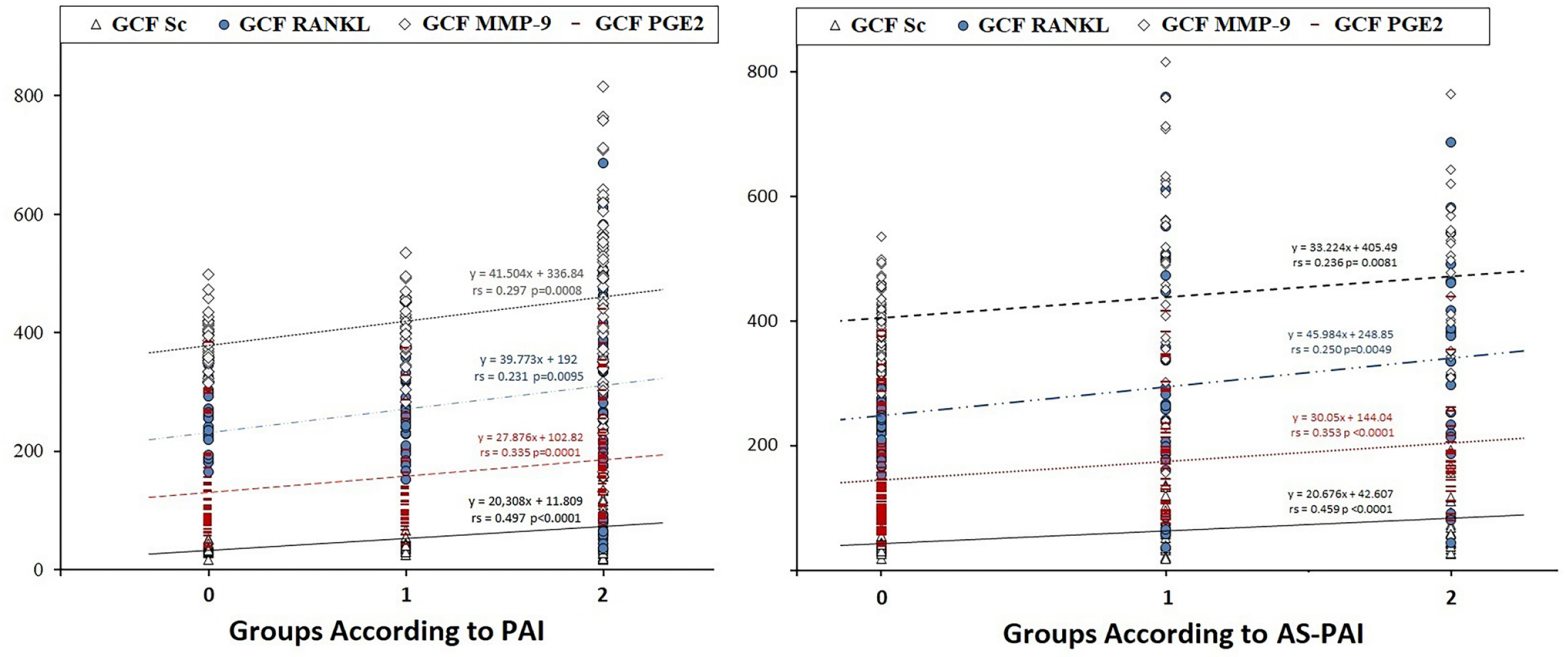
Correlation between PAI and GCF sclerostin, RANKL, MMP-9, and PGE2

Low-to-moderate positive correlation was found between PAI-AP severity and GCF sclerostin, GCF RANKL, GCF MMP-9, and PGE2 levels (rs = 0.497, 95% CI: 0.3471 to 0.6216, *p* < 0.0001; rs = 0.231, 95% CI: 0.05264 to 0.3954, *p* = 0.0095; rs = 0.297, 95% CI: 0.1235 to 0.4539, *p* = 0.0008; and rs = 0.335, 95% CI: 0.1640 to 0.4861, *p* < 0.0001; respectively) (Fig. 3). However, since the correlation between PAI-AP severity and GCF RANKL and GCF MMP-9 was less than 0.30, it was considered statistically insignificant. A statistically significant low positive correlation was found between AS-PAI (abscess score) and OHI score (rs = 0.4605, 95% CI: 0.3052–0.5920, *p* < 0.0001). Low-to-moderate positive correlation was found between AS-PAI and GCF sclerostin, GCF RANKL, GCF MMP-9, and PGE2 levels (rs = 0.459, 95% CI: 0.3031–0.5905, *p* < 0.0001; rs = 0.250, 95% CI: 0.073–0.413, *p* = 0.0049; rs = 0.236, 95% CI: 0.058–0.400, *p* = 0.0081; and rs = 0.353, 95% CI: 0.1839–0.5017, *p* < 0.0001, respectively). However, since the correlation between PAI-abscess score and GCF RANKL and GCF MMP-9 was less than 0.30, it was considered insignificant.

**Fig. 4:**
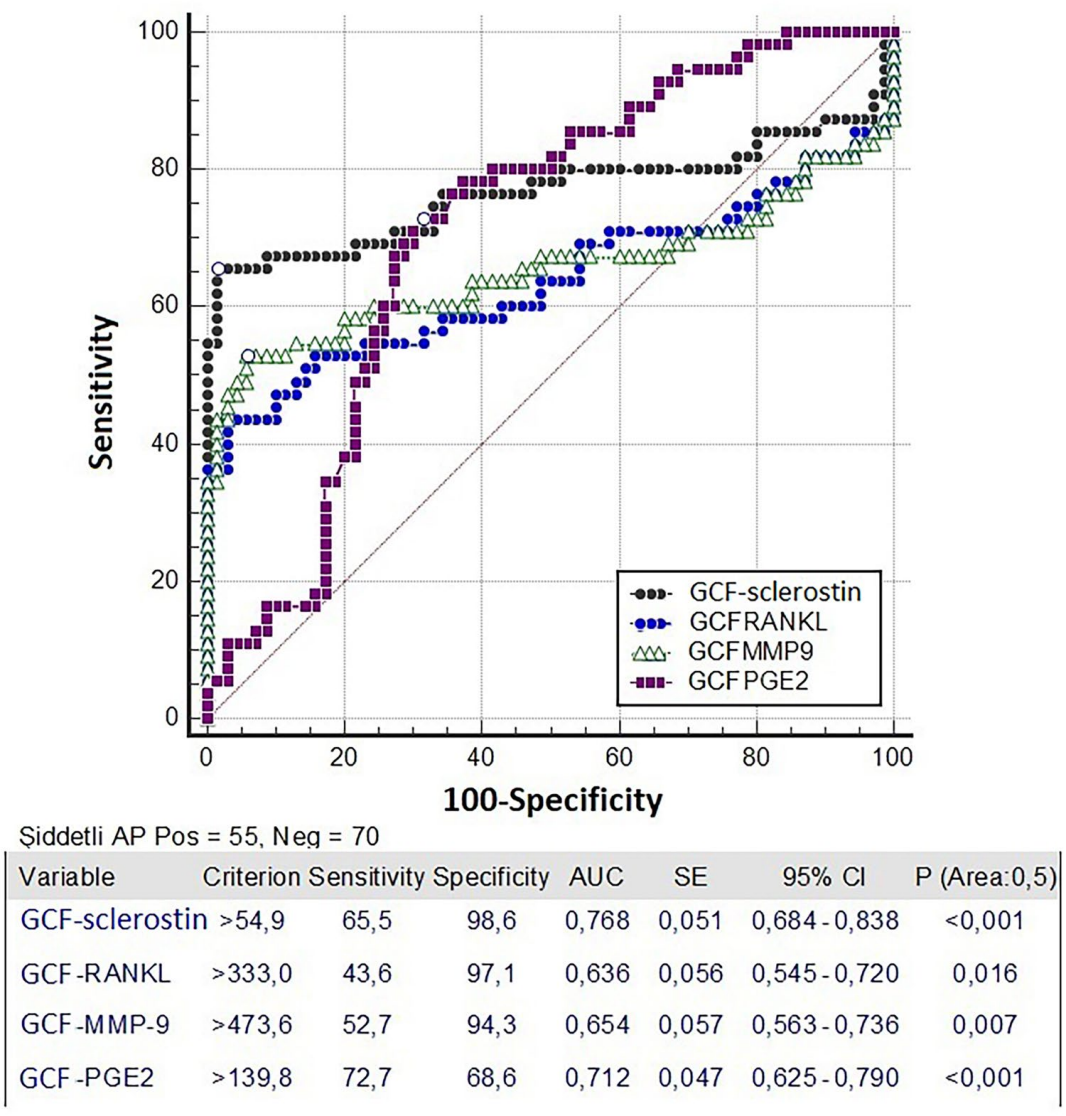
Comparison of 4 parameters with ROC analysis

In the ROC analysis performed to evaluate the diagnostic performance of GCF and serum biomarkers in identifying AP abscess formation, the GCF sclerostin test was found to be at the best threshold value (> 54.9 ng/mL), with sensitivity and specificity values of 65.5% and 98.6%, respectively, and an AUC of 0.768 (*p* < 0.0001). At the best threshold for GCF RANKL (> 333 ng/L), sensitivity and specificity were 43.6% and 97.1%, respectively, with an AUC of 0.636 (*p* = 0.016). At the best threshold for GCF MMP-9 (> 473.6 ng/mL), sensitivity and specificity were 52.7% and 94.3%, respectively, with an AUC of 0.654 (*p* = 0.007). At the best threshold for GCF PGE2 (> 48 ng/L), sensitivity and specificity were 72.7% and 68.6%, respectively, with an AUC of 0.712 (*p* < 0.001) (Fig. 4). Among serum tests, at the best threshold for serum sclerostin (> 10.62 ng/mL), sensitivity and specificity were 70.9% and 58.6%, respectively, with an AUC of 0.640 (*p* = 0.005). At the best threshold for serum PGE2, (> 48 ng/L), sensitivity and specificity were 52.7% and 70.0%, with an AUC of 0.627 (*p* = 0.005). Accordingly, since GCF RANKL, GCF MMP-9, serum sclerostin, and serum PGE2 had AUC values of < 0.70, they were deemed unable to determine abscess formation in AP. Moreover, when Wians et al.’s performance criteria are taken into consideration—namely, that the sum of sensitivity and specificity must be > 170 in order for a laboratory test to be used alone in clinical diagnosis—none of the above-mentioned tests were considered sufficient for determining abscess formation in AP, despite the significant diagnostic performances demonstrated by ROC analysis [31].

In sum, statistical analysis indicated that GCF sclerostin and GCF PGE2 levels are both closely related to abscess formation in AP and can be used independently to determine the severity of AP and bone resorption identified by PAI. In other words, GCF sclerostin and PGE2 levels may be considered to be predictive markers of AP-related abscess formation. Furthermore, when used in combination, OHI score, GCF sclerostin, GCF MMP-9, and GCF PGE2 levels can predict the severity of AP-related abscess formation with approximately 80.8% accuracy. For this reason, using sclerostin and PGE2 tests in GCF alone or more effectively together, rather than serum tests, will be useful in the diagnosis and follow-up of bone resorption (abscess formation), which is the severe form of AP.

## Discussion

Chronic apical periodontitis (AP) is an inflammatory response to the occupation of the root canal system by pathogens and their toxins. Although it is the most common tooth-related inflammatory lesion, AP is usually asymptomatic, and the burden of this endodontic disease is probably underestimated or even unknown. A diagnosis of AP usually requires a combination of clinical (e.g., pain, tenderness to percussion, and palpation) and radiological criteria. Given that radiological imaging alone cannot conclusively identify the severity of AP lesions, there is still a need for simple laboratory parameters that can support these criteria [32]. This study evaluated whether or not serum and GCF levels of sclerostin, RANKL, MMP-9, and PGE2—all biomarkers and proteins with important roles in the pathophysiology of AP—could be used in the diagnosis and follow-up of AP lesions of different severity.

Sclerostin is a biologically active protein involved in bone and tooth metabolism. Produced by mature osteocytes in mineralized bone, this known negative regulator of bone mass and osteoblast differentiation antagonizes the Wnt/β-catenin signaling pathway that plays an important role in osteoblast biology [9, 33]. Inflammation triggered by microbial infections, nonbiological vital formations, and the compressive mechanical effects of orthodontic manipulation can accelerate resorption of the alveolar bone, periodontal ligament, and tooth roots. Several studies have suggested that sclerostin plays a role in accelerating this inflammation and resorption by increasing the RANKL/OPG ratios [34, 35], thereby disrupting the odontoblastic–cementoblastic system responsible for the protection and repair of dentin and tooth roots. This hypothesis is supported by our finding of elevated levels of sclerostin and RANKL in the GCF and serum of patients with periapical bone resorption in connection with severe AP (AS-PAI 3). Furthermore, we found relatively higher concentrations of sclerostin and RANKL in the GCF, as compared to serum, of severe AP patients. We attributed this finding to both the limited area of inflammation (at the tooth root) and the large size of sclerostin and RANKL molecules, which limits their ability to penetrate into the blood. As a protein molecule, sclerostin has a tendency toward hydrophilicity. Studies have shown that sclerostin is secreted from bone tissue and exerts a local/paracrine, rather than endocrine, effect on bone, passing into the bloodstream in limited amounts and thus present in low concentrations in serum under normal conditions [33, 36]. However, changes in sclerostin levels have been observed under certain situations, including some pathological conditions. While this may reflect a systemic effect of sclerostin, additional research is needed before any conclusions can be drawn.

Cellular production of sclerostin can be stimulated by infection, trauma, compression-induced inflammation, and the ischemic-hypoxic state caused by induration. This coincides with the negative role of sclerostin in bone mass. All these results show that sclerostin, like the MMP-9 molecule, plays an important role in the remodeling of the alveolar bone and pulp tissue through osteogenic and osteoclastic processes [4, 37]. MMPs, like sclerostin, are the main enzymes involved in extracellular matrix remodeling. MMPs can operate intracellularly, as well as activating growth factors, cell surface receptors, and adhesion molecules [38]. MMP-9 is secreted by dendritic, hematopoietic, macrophage, neutrophil, fibroblast, and lymphocyte cells [38]. Both MMP-2 and MMP-9 have been shown to be responsible for the degradation of Type-IV collagen in the basement membrane [37, 38]. While found in very small amounts in normal tissue, studies have found high levels of MMP proteins, especially MMP-2 and MMP-9, in areas of tension and compression in teeth undergoing orthodontic treatment [39]. Although our study found no direct relationship between sclerostin and MMP-9, the fact that high levels of sclerostin and MMP-9 levels were found together in GCF suggests the possibility of a complex network of inter-relationships between these two proteins. These two proteins may affect each other through various factors, but more research is needed to better understand the molecular mechanisms involved in this interaction. The results of binary regression analysis that indicated GCF sclerostin and GCF MMP-9 together have a significant effect on AP-related abscess formation is further evidence of a relationship between these two bioactive molecules in terms of bone resorption. Increases in MMP-9 levels in connection with inflammatory conditions and bone injuries may be associated with sclerostin regulation, given that sclerostin, like MMP-9, can regulate cell migration, tissue remodeling, and inflammation through its role in the breakdown of the extracellular matrix [40]. The fact that significant differences among groups were observed in MMP-9 levels in GCF but not in serum was also attributed to the local release of MMP-9 and the limited ability of this large protein to enter into systemic blood circulation.

In our study, statistically significant but low correlations were found between GCF RANKL and GCF MMP-9 levels and both PAI-AP and AS-PAI severity (*r* < 0.30). Although this finding suggests that biomarkers do not show a direct and strong relationship, correlation analysis is not the only indicator of the relationship between two variables. Moreover, correlation analysis only measures linear relationships. It cannot evaluate more complex, indirect or multifactorial interactions between variables. Therefore, the role of GCF RANKL and GCF MMP-9 in chronic AP may be due to different regulatory mechanisms of immune response and bone metabolism, rather than direct interaction. In addition, we believe that evaluating the dynamic changes of these markers over time together with other inflammatory markers in future studies may provide a comprehensive perspective.

Research conducted over the past two decades has identified a number of cytokines, including the TNF family, that are involved in the control of bone formation and destruction. These molecules are understood to affect RANK–RANKL and OPG, which stimulate bone resorption [13, 14]. High amounts of TNF- α, IL-1, IL-6, and PGE2 have been detected in the GCF of patients with periodontal disease [16, 17], indicating that local inflammatory cytokines also play an active role in periodontal disease-related pathologies. These findings suggest that both local and systemic cytokines can be used in the diagnosis and follow-up of periodontal and periapical diseases. At the local level, IL-1 causes endothelial adhesion of leukocytes, stimulates lymphocytes and neutrophils, activates the production of prostaglandins, inhibits bone formation, and enhances bone resorption [1]. PGE2 is one of a number of prostaglandins synthesized in all body tissues in response to physical, chemical, mechanical, immunological, or neurohormonal stimuli. PGE2 is understood to cause an increase in pulp vascular permeability, and it is considered to be the prostaglandin most involved in bone resorption. However, despite its powerful, multifunctional role in the regulation of bone metabolism, PGE2 activity occurs only at the area of synthesis [41]. In vitro studies have demonstrated that PGE2 stimulates bone resorption and increases locally at sites of bone resorption during inflammation [41]. PGEs cause morphological changes in osteoclasts and osteoblasts via cAMP and calcium through increased intracellular levels [42]. Exogenous application of PGE2 has also been reported to increase mRNA synthesis and protein secretion of RANKL [43]. These findings suggest that the changes in periodontal ligament cells observed after exposure to mechanical stress are dependent on PGE2 [41–43]. In a study in which PGE2 concentrations in periapical exudates were determined by radioimmunoassay, higher PGE2 levels were detected in periapical exudates of teeth with radiopaque areas compared to those without radiopaque areas, indicating that high PGE2 levels are an indicator of acute inflammation in the periapical lesion and are produced locally [44]. It also suggested that the PGE2 concentration in periapical exudates of patients with periapical periodontitis is related to disease activity. In this study, both serum and GCF samples were used to biochemically investigate the effects of PGE2 on bone metabolism and root resorption. For this purpose, PGE2 was measured simultaneously using GCF and serum samples of patient groups formed according to PAI scoring. The present study found serum and GCF PGE2 levels to be higher in cases of severe AP with apical resorption; however, the increases were more pronounced in GCF as compared to serum. This can most likely be attributed to the lipophilic nature of the PGE2 molecule, which allows it to move from cells into the bloodstream with relative ease. This finding was different from the situation detected for serum RANKL and MMP-9.

In general, a single parameter is not sufficient for disease diagnosis, which requires a number of additional parameters to support a main parameter. Regression analysis performed in this study determined that a model consisting of OHI score, GCF sclerostin, GCF MMP-9, and GCF PGE2 tests could predict abscess formation related to AP severity with an accuracy of approximately 80.8%. ROC analysis indicated that GCF sclerostin and GCF PGE2 tests had sufficient diagnostic performance to be used in the diagnosis of abscess formation in AP. However, as stated by Wians et al., for a laboratory test to be used alone in clinical diagnosis, the sum of its sensitivity and specificity performance must be > 170 [31]. When this criterion is taken into consideration, none of the parameters examined could be said to have sufficient performance for use alone, despite the considerable diagnostic performance demonstrated by certain parameters in predicting severe AP according to ROC analysis. Accordingly, either a quadruple regression model consisting of OHI score, GCF sclerostin, GCF MMP-9, and GCF PGE2 or the combined measurements of GCF sclerostin and GCF PGE2 were deemed more appropriate for predicting AP-related resorption in the periapical region. Therefore, once the threshold values of sclerostin and PGE2 are standardized through larger studies such as cohort, these biomarkers in GCF samples obtained at diagnosis and post-treatment could be valuable within a clinical workflow for identifying AP status and monitoring its management.

Previous studies have examined the use of PGE2 in determining postoperative pain and in connection with clinical symptoms [45–47]. While ours is the first study correlating PGE2 levels with radiographic findings, in line with previous studies, we also propose the use of PGE2 for AP diagnosis and follow-up. Moreover, while the role of sclerostin has been previously identified in other forms of periodontal disease [37], our study is the first to examine the relationship between sclerostin and AP. This study showed that the possible cause of bone resorption observed in AP could be explained by the interaction between PGE2, which increases osteoclast activation, and sclerostin, which regulates bone metabolism. To summarize the results, PGE2 promotes osteoclastogenesis by activating the RANKL pathway, while sclerostin suppresses bone formation by inhibiting the Wnt/β-catenin signaling pathway; thus, both molecules may contribute to the progression of bone destruction.

## Study limitations

The sample size in this study may limit the generalizability of the findings. Therefore, it would be beneficial to conduct a larger, multicenter study to confirm these results in different populations and settings. Although this study attempted to control for basic demographic factors such as age, gender, and BMI, and oral hygiene using the OHI-S index, other variables such as smoking, diet, systemic inflammatory conditions, and medication use may affect biomarker levels. This study was conducted on a specific patient population visiting a single dental clinic. This single-center design may increase bias to some extent and may partially limit the external validity and reproducibility of our results. In addition, the cross-sectional nature of this study may reduce the strength of a causal relationship between the observed biomarker levels and the progression of apical periodontitis compared to prospective or meta-analysis studies. Therefore, longitudinal studies following patients over time will be necessary to determine whether changes in sclerostin, PGE2, RANKL, and MMP-9 levels precede or result in AP severity and abscess formation.

## Conclusions

GCF levels of sclerostin, a powerful inhibitor of bone formation, and PGE2, which regulates many biological responses of cells, including inflammation, pain, and fever, were both shown to have a close relationship with the PAI abscess scores used to determine the severity of AP. However, no direct relationship was found between sclerostin and PGE-2 levels. Moreover, while neither of these biomarkers alone can predict AP-related bone resorption, when used together, GCF sclerostin and PGE-2 levels were shown to be effective in diagnosing bone resorption in AP and monitoring its progression.

## Data Availability

The data applied in this study are reasonably available from the corresponding author.
